# Anillin Phosphorylation Controls Timely Membrane Association and Successful Cytokinesis

**DOI:** 10.1371/journal.pgen.1006511

**Published:** 2017-01-12

**Authors:** Hyunjung Kim, James M. Johnson, Robert F. Lera, Sarang Brahma, Mark E. Burkard

**Affiliations:** University of Wisconsin Carbone Cancer Center and Department of Medicine, Hematology/Oncology, University of Wisconsin-Madison, Madison, Wisconsin, United States of America; UNC-Chapel Hill, UNITED STATES

## Abstract

During cytokinesis, a contractile ring generates the constricting force to divide a cell into two daughters. This ring is composed of filamentous actin and the motor protein myosin, along with additional structural and regulatory proteins, including anillin. Anillin is a required scaffold protein that links the actomyosin ring to membrane and its organizer, RhoA. However, the molecular basis for timely action of anillin at cytokinesis remains obscure. Here, we find that phosphorylation regulates efficient recruitment of human anillin to the equatorial membrane. Anillin is highly phosphorylated in mitosis, and is a substrate for mitotic kinases. We surveyed function of 46 residues on anillin previously found to be phosphorylated in human cells to identify those required for cytokinesis. Among these sites, we identified S635 as a key site mediating cytokinesis. Preventing S635 phosphorylation adjacent to the AH domain disrupts anillin concentration at the equatorial cortex at anaphase, whereas a phosphomimetic mutant, S635D, partially restores this localization. Time-lapse videomicroscopy reveals impaired recruitment of S635A anillin to equatorial membrane and a transient unstable furrow followed by ultimate failure in cytokinesis. A phosphospecific antibody confirms phosphorylation at S635 in late cytokinesis, although it does not detect phosphorylation in early cytokinesis, possibly due to adjacent Y634 phosphorylation. Together, these findings reveal that anillin recruitment to the equatorial cortex at anaphase onset is enhanced by phosphorylation and promotes successful cytokinesis.

## Author Summary

Human diseases such as cancer and congenital trisomies arise from loss of genetic material during cell division. Yet in most divisions, cells preserve their genetic integrity by strict coordination of cell membrane cleavage (cytokinesis), with accurate separation of genetic material (mitosis). Thus, understanding how mitosis and cytokinesis are coordinated can provide insight into human disease. Anillin is one central integrator of cytokinesis that is recruited in a ring-like structure on the membrane of dividing cells. Protein phosphorylation is a common mechanism regulating timing of events in mitosis; although 46 phosphorylation sites have been mapped on anillin, their functional significance is unknown. Here, we evaluated the effect of blocking anillin phosphorylation on human cell division. Surprisingly, most phosphorylation events appeared dispensable for cytokinesis in the assay used. By contrast, phosphorylation of S635 is important for early recruitment of anillin to the midzone membrane, furrow stabilization and efficient cytokinesis. Our findings highlight a central mechanism regulating the timing of human cytokinesis.

## Introduction

In cytokinesis, cells assemble and stabilize an actomyosin ring between segregated chromosomes to generate daughter cells. The positioning of the cleavage furrow is controlled by negative signals from astral microtubules and positive signals established from the central spindle [1, 2]. At anaphase onset, inactivation of cyclin-dependent kinase 1 (Cdk1) triggers recruitment of centralspindlin to the central spindle and adjacent equatorial cell membrane [3–5]. Centralspindlin recruits the RhoGEF, epithelial cell transforming sequence 2 (Ect2), to locally activate the small GTPase RhoA [2, 4, 6] which specifies localization of the contractile actomyosin ring [7]. Temporal recruitment and activation of the centralspindlin apparatus depends on phosphorylation, including Cdk1-dependent phosphorylation sites on mitotic kinesin-like protein 1 (MKLP1) and Ect2, lost at anaphase onset [3, 6], and anaphase-specific recruitment of Plk1 through phosphorylation of protein regulating cytokinesis 1 (PRC1) [8]. Phosphorylation is likely to regulate additional events in cytokinesis.

**Fig 1:**
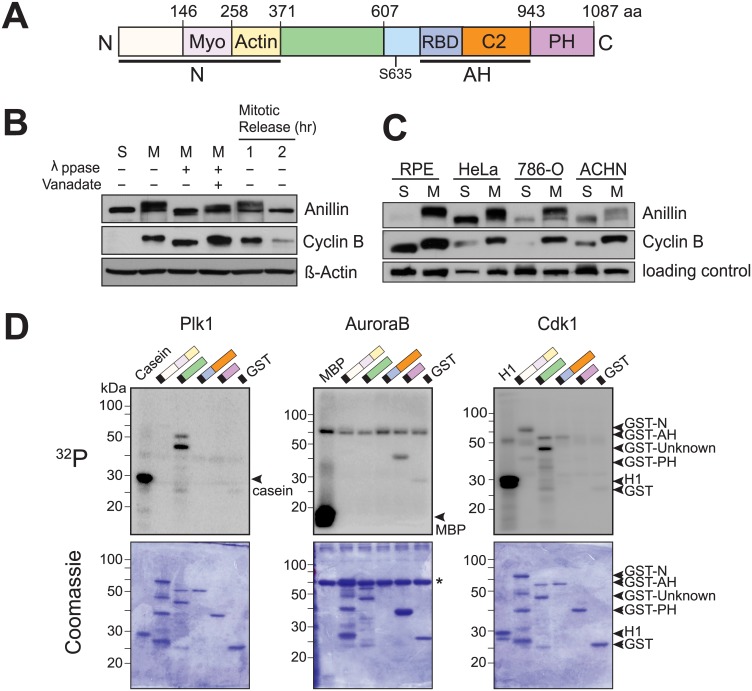
Anillin is highly phosphorylated in mitosis. (A) Schematic of anillin domains (Myo = myosin binding; RBD = Rho Binding Domain; AH = anillin homology; PH = pleckstrin homology). (B) Immunoblot analysis of electrophoretic shift of anillin. HeLa cells were arrested using nocodazole (0.2 μg/ml) for 12 h (M) and released into fresh drug-free medium for the indicated time. S phase cells by double thymidine block serves as control. Cyclin B loss confirms mitotic exit. λppase = lambda phosphatase, vanadate = sodium orthovanadate (Na_3_VO_4_). (C) The mitotic hyperphosphorylation of anillin is a common feature in various cell lines. RPE1 is a non-transformed human retinal pigment epithelial cell line; 786-O and ACHN are human renal cancer cell lines. Loading control: a non-specific band detected by anillin antibody. (D) In vitro kinase assays with indicated kinases and GST-tagged anillin fragments. Protein phosphorylation is visualized by autoradiography (^32^P, top panel) and equal protein loading by Coomassie blue staining (bottom panel). *: Bovine serum albumin.

Anillin is a key scaffold protein linking the actomyosin ring to the equatorial membrane [9–11]. Anillin binds myosin and F-actin at the N-terminus, and has anillin homology (AH) and pleckstrin homology (PH) domains at the C-terminus (Fig 1A). Anillin’s myosin and F-actin binding domain are required for organization of the actomyosin ring [12, 13]. The AH domain of anillin binds RhoA and shares homology with Rhotekin, a RhoA-GTP binding domain [10]. *Drosophila* anillin also binds to RacGAP50C, a homologue of MgcRacGAP, suggesting that it may couple the central spindle to a contractile ring [14, 15]. The C-terminal PH domain is important for recruitment of anillin to the equatorial membrane. Recently, a cryptic C2 domain within the AH domain was discovered and, with adjacent PH domain, promotes efficient recruitment to the membrane [16]. Thus, anillin is a hub for midzone membrane regulators and effectors of cytokinesis. Highlighting its required role in these processes, anillin depletion results in cytokinesis failure [13, 17, 18]. Despite its importance, poorly defined mechanisms restrict anillin to operate specifically at cytokinesis and at the equatorial membrane.

Anillin localization varies with the cell cycle. In interphase, anillin primarily localizes to the nucleus [9, 12], but upon entry into mitosis it re-localizes uniformly to the cell cortex. At anaphase onset, anillin is lost from the poles and concentrates at the equatorial zone, prior to onset of cytokinesis [19]. The loss adjacent to the poles is explained in part by Ran-GTP signals which emanate from chromatin [20]. One candidate for anillin recruitment is concentration of its preferred lipid phosphatidylinositol 4,5-bisphosphate (PI(4,5)P_2_) at the equatorial membrane [21]. Locally activated RhoA promotes anillin recruitment to the equatorial membrane and anillin stabilizes RhoA against extraction with trichloroacetic acid (TCA) [10, 18, 19]. Although these may be sufficient to specify timely recruitment of anillin, additional regulatory mechanisms of temporal-spatial control could reinforce these signals.

Protein phosphorylation is a common mechanism of regulating localization and function in mitosis. Anillin displays phosphorylation-induced band retardation in SDS-PAGE [22] and large-scale phosphoproteomics identified numerous phosphorylation sites [23, 24]. In fission yeast, the Polo-like kinase Plo1 binds to and phosphorylates an anillin-like protein Mid1, facilitating contractile ring assembly [25]. Similarly, phosphorylation sites of *S*. *cerevisiae* anillin homolog, Bud4, by Cdk1 promotes cytokinesis [26]. However, human anillin is divergent from its yeast counterparts, and several phosphorylation sites are not conserved, suggesting that the regulation may likewise differ. We hypothesized that phosphorylation of anillin governs its required function in human cytokinesis.

Here we report that human anillin is indeed phosphorylated in mitosis, and that phosphorylation is required for proper cytokinesis. To evaluate functional significance of phosphorylation, we used a stringent assay to comprehensively evaluate non-redundant phosphorylation events on anillin. Surprisingly, most phosphorylation events are dispensable for cytokinesis. However, we identify phosphorylation of a single residue, S635 as a key determinant of anillin localization and function. This phosphorylation site controls its ability to efficiently concentrate on the equatorial plasma membrane, and maintain a stable cytokinetic furrow. This finding suggests that phosphorylation at this site controls timely equatorial accumulation of anillin required for cytokinesis.

## Results

To investigate anillin phosphorylation we first characterized how it is modulated in human mitosis. In mitotic HeLa extracts, anillin migrated slowly on SDS-PAGE compared to its S-phase counterpart (Fig 1B). This shift was reversed by Lambda phosphatase, indicating the retardation is attributable to phosphorylation. Consistent with phosphorylation being specific to mitosis, the slow-migrating band diminished upon mitotic exit (Fig 1B, right lanes). These observations were not specific to HeLa; similar mitotic shifts were observed for anillin in other cancer cells and non-transformed human cells (Fig 1C). We conclude that anillin is phosphorylated significantly in human mitosis.

To identify kinases that might be responsible for mitotic phosphorylation of anillin, we purified recombinant GST-tagged fragments and performed in vitro kinase assays with the mitotic kinases polo-like kinase 1 (Plk1), Aurora B, and Cdk1. Under these conditions, all phosphorylated anillin, though on different domains: Plk1 and Cdk1 phosphorylated the 371–607 fragment, whereas Aurora B preferred the PH domain (Fig 1D). To test if one kinase is primarily responsible for the electrophoretic mobility shift, we employed specific inhibitors of Plk1, Aurora B, and Cdk1. Inhibition of Cdk1 with RO-3306 restored fast electrophoretic mobility not seen with other inhibitors (S1 Fig). Thus phosphorylation events that shift anillin mobility are governed directly or indirectly by Cdk1, although these findings do not exclude possible roles by Plk1 or Aurora B, as some phosphorylation events might not significantly alter shift. Additionally, other kinases could operate on anillin, such as Citron or Rho kinases, which have a known role in cytokinesis [27]. We conclude that anillin is phosphorylated in mitosis and is regulated by mitotic kinases, consistent with a possible regulatory role in cytokinesis.

**Fig 2:**
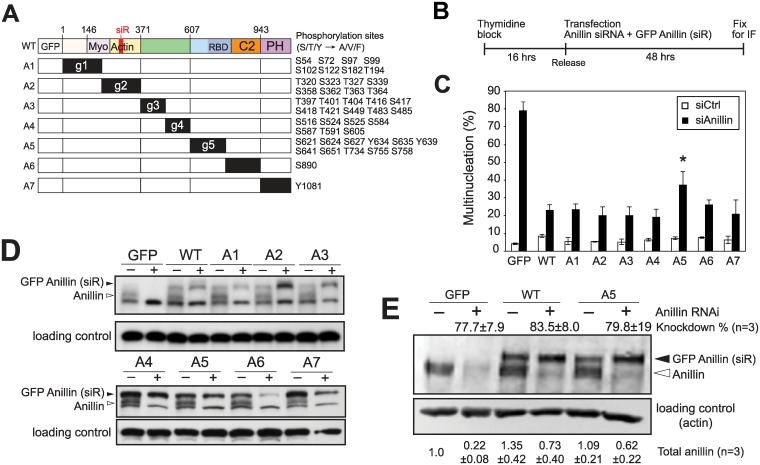
Efficient cytokinesis requires phosphorylation in or near the Anillin Homology (AH) domain. (A) Schematic of GFP-tagged non-phosphorylatable anillin constructs with siRNA-resistant (siR) silent mutations. Non-phosphorylatable mutants were generated by either gBlock/Gibson assembly (A1-A5) or site-directed mutagenesis (A6 and A7). Mutated phosphorylation sites of each construct were listed on the right (Ser/Thr/Tyr mutated to Ala/Val/Phe, respectively). (B-D) RNAi-mediated rescue experiment with non-phosphorylatable mutants. (B) Experimental protocol. HeLa cells were arrested by thymidine for 16 h, followed by a 1 h release to fresh medium and then transfected with anillin siRNA and the indicated GFP anillin constructs. After 48 h, cells were fixed and processed for immunofluorescence. (C) Multinucleation in GFP-positive interphase cells 48 h after transfection. Error bars, mean ± s.e. of three experiments (n > 200 cells each). Statistical comparison between WT and A5 was made with a two-tailed Student’s t-test (*p< 0.05). (D) Immunoblot analysis of protein extracts prepared from B. Endogenous anillin and transgenic GFP anillin are indicated by open and filled arrowheads, respectively. The loading control is a ~300 kDa non-specific band detected with anillin antibody. (E) Quantitative immunoblotting revealing degree of knockdown and addback in control and A5 conditions. ± SD is shown.

As multiple mitotic kinases can phosphorylate anillin, we focused on residues known to be phosphorylated without kinase bias. From two phosphorylation site databases, we identified 46 sites, the majority discovered by phosphoproteomic mass spectrometry [23, 24]. These 46 sites (Ser, Thr or Tyr) were fragmented into 7 subdomains, and we made constructs for each, replacing all with non-phosphorylatable residues (Ala, Val or Phe, respectively) (Fig 2A). For the mutants containing a large number of phosphorylation sites (A1-A5), we employed the Gibson assembly method to assemble blocks of synthetic double-stranded DNA [28]. To study the functions of these phosphorylation events, we optimized an RNAi knockdown and transgene rescue assay (Fig 2B). This assay was developed under conditions to establish the most robust difference between the GFP and WT controls to allow functional assessment of anillin mutants. In an asynchronous population of cells fixed after 48 h of RNAi treatment, 79% of GFP-positive cells were multinucleate. This phenotype was efficiently suppressed by coexpression of siRNA-resistant wild-type anillin (Fig 2C and 2D). Under these conditions, the GFP-tagged constructs in panel A were assessed for their ability to restore cytokinesis. Strikingly, most non-phosphorylatable constructs suppressed multinucleated cells similar to wild type. However, the A5 mutant had a two-fold increase in multinucleation, despite efficient expression. To evaluate the efficacy of knockdown/addback under these conditions, we performed quantitative immunoblotting with infrared secondary antibodies (Fig 2E), revealing that knockdown was ~80% effective, and that transfection added back anillin below endogenous levels. We conclude that most phosphorylation sites are dispensable in this assay, but that one or more of these sites in the g5 domain play a crucial non-redundant role in successful cytokinesis.

**Fig 3:**
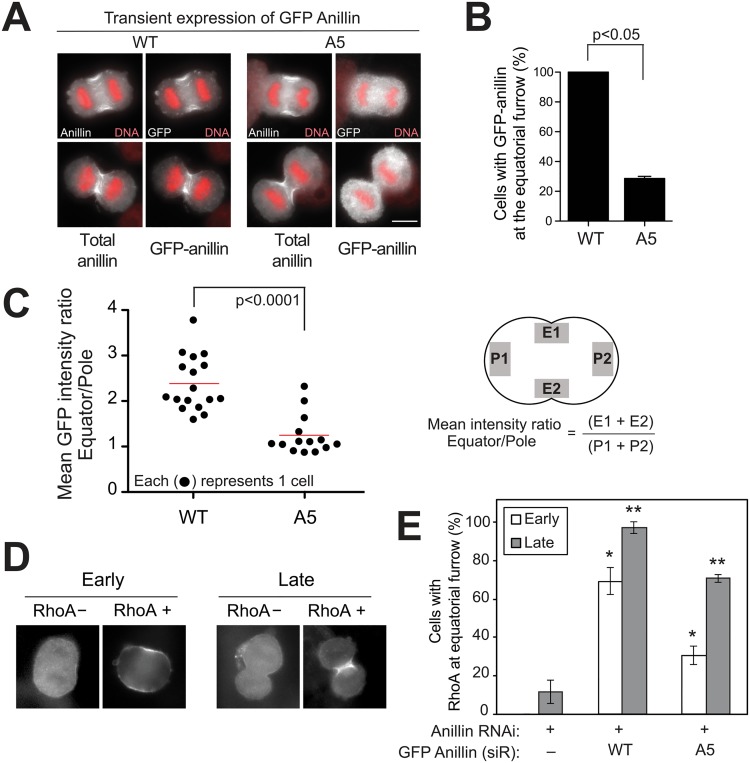
Phospho-deficient A5 mutant has impaired equatorial localization and is unable to fix RhoA. (A) IF analysis of transiently expressed A5 in formaldehyde-fixed anaphase cells. GFP-A5 and endogenous anillin were detected using anti-GFP and anillin antibodies, respectively. (B) Quantification of furrow localization of GFP-anillin and GFP-A5. Error bars, mean ± s.e. from three experiments (n > 30 cells each). p<0.05 by Student’s t-test. Scale bar, 10 μm. (C) Mean intensity ratio (GFP fluorescence at the equatorial cortex:pole) of individual cells were plotted for each GFP anillin construct. Red bars indicate median values. p<0.0001 by Student’s t-test. Right: diagram showing locations of regions of interest. Note: used cytoplasmic GFP levels at the poles. (D-E) IF analysis of furrow RhoA after rescue experiment with A5. HeLa cells simultaneously transfected with anillin siRNA and the indicated GFP anillin constructs. Transfected cells were enriched in anaphase by monastrol block and release. (D) Representative images of furrow RhoA at either early or late stage of cytokinesis. (E) Quantification of RhoA at equatorial furrow in TCA-fixed anaphase cells. Error bars, mean ± s.e. from three experiments (n > 20 cells each; * and **, p<0.05).

Because anillin predominantly localizes at the cleavage furrow in anaphase, we tested if the A5 mutant impairs localization to the furrow. To do this, HeLa cells were transiently transfected with either GFP-tagged wild-type or A5 anillin, enriched at anaphase and assessed for localization. Endogenous anillin was used as internal control to verify that each cell is evaluated at the time proper for anaphase localization. Indeed, we found that A5 anillin remains diffused throughout the cytoplasm in early and late anaphase, wherein endogenous anillin concentrates at furrow (Fig 3A). This phenotype was scored in two independent ways: a cell population scored by a blinded observer where only cells with strong furrow localization was counted (Fig 3B), and an intensity analysis in which the GFP fluorescence at the equatorial membrane was normalized to the cytoplasmic levels near poles in a single cell (Fig 3C). Both analyses confirmed impaired recruitment of A5 anillin at the furrow. We conclude that the A5 construct, with 11 nonphosphorylatable mutations, is not efficiently recruited to the equatorial midzone on anaphase onset. RhoA is a central regulator of furrow formation [29, 30] and its TCA-fixable cortical pool is dramatically reduced by loss of anillin [10]. To test if A5 was able to stabilize active RhoA, cells were fixed/extracted with TCA [31]. As expected, fixed cortical RhoA is significantly reduced at both early and late anaphase cells expressing A5 anillin (Fig 3D and 3E). These findings suggest that A5 anillin has impaired equatorial recruitment and is unable to fix cortical RhoA at the furrow.

**Fig 4:**
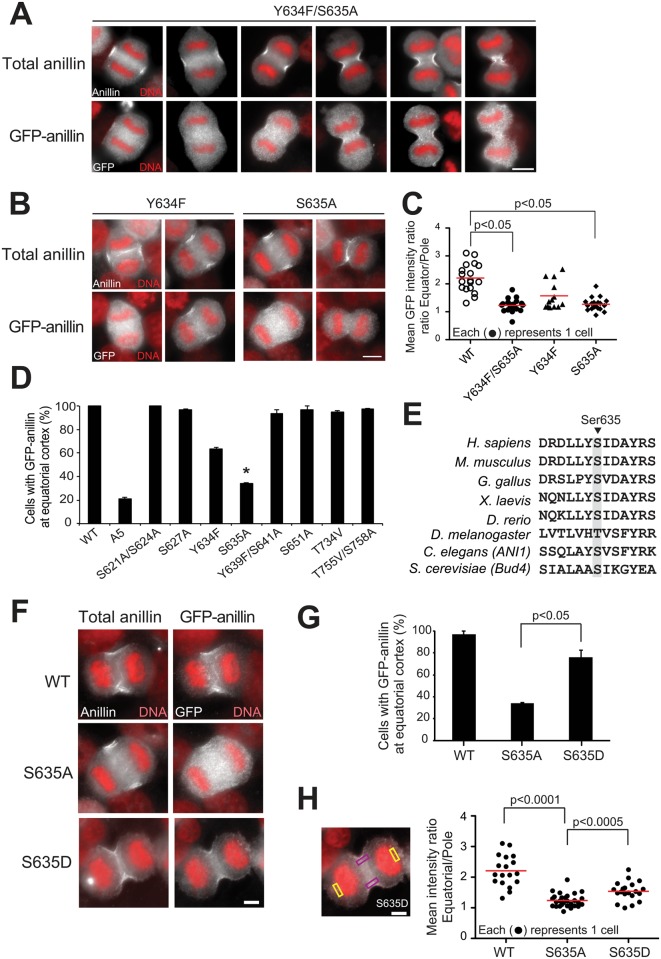
Identification of Ser635 as a critical site for the equatorial localization of anillin at furrow. (A) IF analysis of transiently expressed GFP-anillin Y634F/S635A double mutant. GFP anillin mutants and endogenous anillin were detected using anti-GFP and anillin antibodies, respectively. (B) Localization of Y634F and S634 mutant anillin by IF. (C) Mean intensity ratio of GFP fluorescence at the equatorial cortex:pole for the indicated GFP anillin mutants. Red bars indicate median values. P values by Student’s t-test. (D) Quantification of GFP anillin mutants at equatorial furrow. Summary of 8 single non-phosphorylatable GFP mutants’ localization. Error bars, mean ± s.e. from three experiments. *p<0.05 (E) Sequence alignment of the region surrounding S635, revealing conservation. (F) IF analysis of transiently expressed phosphomimetic anillin, S635D. (G) Quantification of phosphomimetic S635 Anillin furrow localization by observer (H) Quantitative immunofluorescence with representative cell from panel F showing regions quantified in violet (equator) and yellow (pole). Red bars in H indicate median values. P values by Student’s t-test. Scale bars, 5 μm. See also S2 and S3 Figs.

Phosphorylation sites could operate distributively to enhance furrow recruitment of anillin. Alternatively, a single phosphorylation site could be primarily responsible. To test this, we generated single/double non-phosphorylatable mutants for the each of the 11 sites in A5 anillin. Each GFP-tagged mutant was transiently expressed in HeLa cells and analyzed for localization. Most single/double mutants localize properly like their endogenous counterparts (S2 Fig). However, anillin Y634F/S635A behaved like A5, showing a dispersed localization (Fig 4A). To distinguish the relative contributions of Y634 and S635, we tested mutations singly. Both mutants disrupt anillin localization, but S635A had a more marked contribution (Fig 4B–4D). Thus, from 11 sites, we identified S635 as a major residue required for proper anaphase localization of anillin. Moreover, S635 is conserved across metazoans, supporting its role as a critical phosphorylation site for cytokinesis (Fig 4E).

In principle, loss of the hydroxyl group in S635A anillin could disrupt protein function by a mechanism other than preventing phosphorylation. If phosphorylation per se is important, its function could be restored by a phosphomimetic negatively-charged residue at this site. Indeed, anillin S635D concentrated at the equatorial cortex (Fig 4F); this was clearly distinguishable from the one of S635A, as judged by a blind observer assay (Fig 4G) or by quantitative immunofluorescence (Fig 4H), although it did not restore recruitment to the furrow to the extent of wild type. This disrupted localization is specific to anaphase, as anillin S635A localized properly in interphase and metaphase (S3 Fig). Thus, S635 is crucial for anillin localization at the furrow but dispensable for localization at other times. These results support the idea that phosphorylation of S635 is critical for anillin recruitment to equatorial membrane in cytokinesis.

**Fig 5:**
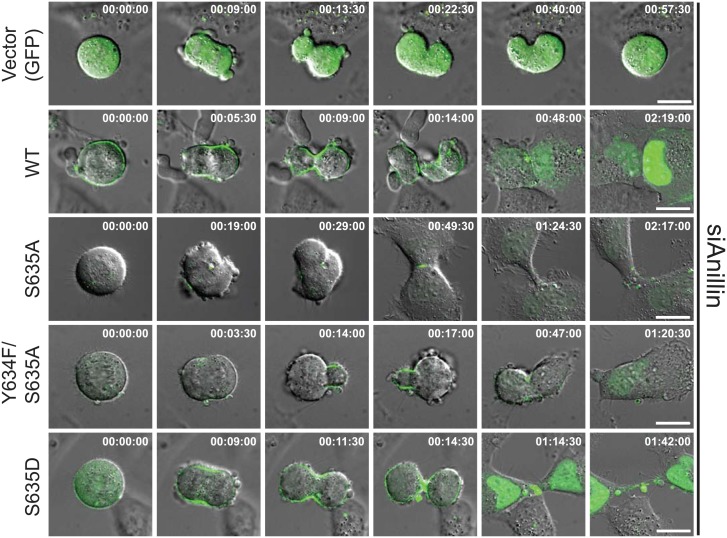
Timelapse videomicroscopy of anillin mutants. To assess contractile rings in live cells, timelapse videomicroscopy was performed in HeLa cells in which endogenous anillin was depleted and the GFP-tagged constructs were transiently transfected. The merge of DIC and GFP are shown. GFP signal is scaled equivalently among all constructs. Times from anaphase onset is reported (hours:min:sec) in the upper right of each panel. Scale bar, 10 μm. See also S4 Fig and Supplementary Videos.

To characterize the cytokinesis defects and membrane recruitment, we performed timelapse videomicroscopy of wild type and mutant anillin constructs after depleting endogenous anillin (Fig 5 and S1–S5 Videos). With depletion of anillin and GFP transfection, we observed marked furrow instability, as expected [10, 13, 19]. This effect was rescued with GFP-wildtype anillin, which was efficiently recruited to membrane and enriched at the furrow by 5–9 minutes after anaphase onset. By contrast, both S635A and double mutant Y634F/S635A were poorly recruited to membrane during mitosis, although some furrow localization is seen. These constructs failed to restore furrow stability and resulted in significant oscillation of furrows. The phosphomimetic, S635D, restores membrane recruitment, enrichment at the cleavage furrow, and stabilization of the mitotic furrow. To assess this in a larger population of cells, we analyzed furrows in timelapse videomicroscopy of HeLa cells expressing mCherry-H2B (S4 Fig). As above, these images reveal transient furrows and then failed cytokinesis to generate a single cell with two nuclei.

**Fig 6:**
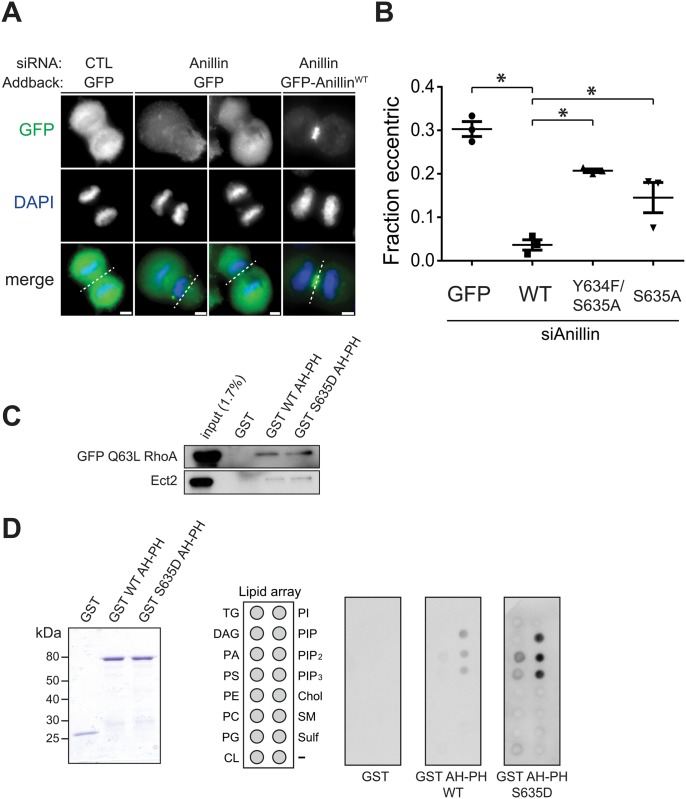
Ser635A anillin exhibits a destabilized furrow, and phosphomimetic does not alter binding to Ect2, RhoA, or selectivity for specific phospholipids. (A) Knockdown/addback revealed eccentric furrows seen in anaphase cells after depletion of anillin. Dashed line marks approximate region of furrow. Scale bar, 5 μm. (B) Asynchronous Hela cells were treated by knockdown/addback and analyzed for furrow location at 48 hours. Fraction of eccentric furrows was quantified in anaphase and telophase cells (n = 3; ≥50 cells per condition). *ANOVA detected differences between wild-type rescue and other conditions. (C) Western blot analysis of pulled down RhoA and Ect2 with GST AH-PH anillin. HeLa extracts from transfected with GFP Q63L RhoA (top panel) or untransfected (bottom) were used in pull-down assays with wild-type or phosphomimetic S635 GST AH-PH. (D) Coomassie blue staining of recombinant GST and GST AH-PH anillin (left panel). GST fusion proteins were expressed in *E*. *coli* and purified using Glutathione Sepharose 4B Fast Flow. Lipid arrays (middle panel) were incubated with the indicated recombinant proteins before being probed by anti-GST (right panel). Key for lipid array: TG = triglyceride; DAG = diacylglycerol; PA = phosphatidyl acid; PS = phosphatidylserine; PE = phosphatidylethanolamide, PC = phosphatidylcholine; PG = phosphatidylglycerol; CL = cardiolipin; PI = phosphatidylinositol; PIP = PI 4-phosphate; PIP_2_ = PI 4,5-bisphosphate; PIP_3_ = PI 3,4,5-triphosphate; Chol = cholesterol; SM = sphingomyelin; Sulf: sulfatide; - = Blank.

To assess frequency of furrow instability with non-phosphorylatable anillin, we developed a fixed-cell assay to identify cells with unstable furrows (Fig 6A and 6B). As expected anillin depletion increased the number of cells with eccentric furrows. Likewise, non-phosphorylatable mutants had increased eccentric furrows compared with wild-type control. Thus, the non-phosphorylatable mutants of anillin are unable to sustain a stable contractile ring, consistent with poor recruitment of these mutants in early anaphase. Together, these data provide high confidence that S635 is important to sustain the mitotic furrow.

We next considered mechanism for the cortex association of phospho-S635 anillin. The AH domain of anillin adjacent to S635 is known to interact with RhoA and Ect2 [10, 32]. Therefore, we reasoned that phopho-S635 anillin might relieve autoinhibition of the AH domain to stabilize its interaction with these interactors. To address this, we performed a pulldown assay from mitotic HeLa extracts with either unphosphorylated (GST WT AH-PH) or the phosphomimetic (GST S635D AH-PH) C-terminal anillin fragment. For RhoA pull down, cells were transfected with constitutively active RhoA (Q63L). However, RhoA and Ect2 were pulled down equally well by the wild-type and phosphomimetic C-terminal anillin fragment (Fig 6C). We conclude that S635 phosphorylation is unlikely to regulate interaction with Ect2 or active RhoA.

A key localization requirement for anillin is phosphatidylinositol phosphate at the plasma membrane, mediated by its C-terminal PH domain [10]. This prompted us to test the effect of phosphorylation at S635 on association with membrane phospholipids. GST-tagged anillin AH-PH variants were incubated with an array of lipids that were immobilized on a support membrane. GST fusions of both wild-type and phosphomimetic AH-PH bound to phosphatidylinositol 4-phosphate PI(4)P, PI(4,5)P_2_, and PI(3,4,5)P_3_. However, there was no observed difference in preference for phospholipids (Fig 6D). Thus the specific mechanism by which phosphorylation regulates anillin recruitment and furrow stability appears to be distinct from its roles in binding RhoA, Ect2, and does not control specificity for binding of the C-terminus to specific phospholipids.

**Fig 7:**
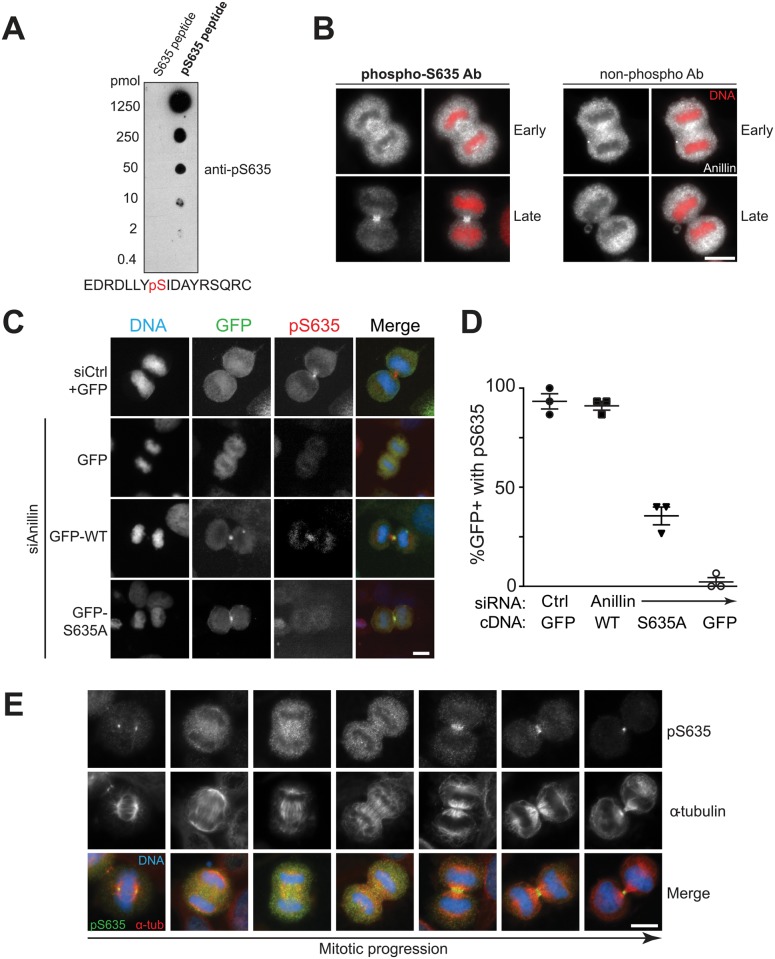
Anillin Ser635 is an in vivo phosphorylation site. (A-D) Validation of phosphospecific antibody raised against pS635. (A) Known amounts of phosphorylated (pS635) or unphosphorylated (S635) peptides were spotted on PVDF and immunoblotted with anti-pS635 antibody to assess phosphoselectivity. Phosphopeptide sequence for immunization; pS635 in red. (B) IF staining of cells in early (top) or late (bottom) anaphase by affinity purified anti-pS635 antibody (phospho-S635 Ab). Antibody purified with non-phosphorylated immunizing peptide (non-phospho Ab) was used as control. Scale bar, 10 μm. (C) Knockdown/addback experiment demonstrating that phospho-anillin staining fails to detect anillin S635A. Scale bar, 5 μm (D) Fraction of GFP positive (GFP+) cells that have localized pS635 signal. (E) Phosphospecific S635 antibody detects anillin at late stage of cytokinesis. Representative images of cells are shown from metaphase to late cytokinesis. Cells were stained by anti-pS635 antibody (green), anti-α-tubulin (red) and DAPI (blue). Scale bar, 10 μm. See also S5–S7 Figs.

To evaluate localization of phosphorylation, we raised a polyclonal antibody against a peptide encompassing phospho-S635, and validated it by several measures. First, dot-blot verified >1000 fold phospho-specificity, as non-phosphorylated peptide was not detected at up to 1250 pmol (Fig 7A). By Western blot of whole cell lysates, pS635 antibody recognized bands from HeLa lysates that matched those detected by anti-anillin antibody and decreased upon anillin depletion (S5 Fig). We were unable to fully validate specificity for pS635 by immunoblot. By contrast, immunofluorescence confirmed reactivity of the phosphospecific antibody at the midbody in late cytokinesis, whereas the non-phosphospecific antibody did not detect an epitope at this site (Fig 7B). Finally, siRNA against anillin/addback revealed that the phosphoepitope is detected with wild-type anillin transfection, but not with S635A (Fig 7C and 7D). We conclude that this antibody specifically detects phosphorylated S635 by immunofluorescence.

Having established the specificity of the pS635 antibody in immunofluorescence, this reagent was used to investigate the dynamics of phosphorylation during cytokinesis. Localized S635 phosphorylation appears in cells after furrowing and persists until late cytokinesis (Fig 7E). Strongly enriched signal is seen at the cortical midzone at late cytokinesis. We performed a more detailed analysis of pS635 signal at different stages of anaphase under different fixation/extraction conditions and confirmed that the signal is only seen in late mitosis (S6 Fig). However, it was puzzling that pS635 is not detected earlier in anaphase when anillin is being recruited to membrane. We considered that the adjacent post-translational modifications could partially interfere with the antibody. Indeed, the antibody fails to detect a doubly phosphorylated peptide at both Y635 and S635 (S7 Fig). We conclude that S635 on anillin is an in vivo phosphorylation site, although our reagent cannot detect early mitotic phosphorylation, possibly due to interference with adjacent post-translational modifications.

## Discussion

Anillin is phosphorylated in mitosis [22], but heretofore, the physiological role of these post-translational modifications has been obscure. We discovered that among 46 phosphorylated residues, S635 is required for efficient anillin recruitment to the furrow and for successful cytokinesis. Several lines of evidence confirm that S635 is important due to phosphorylation at this site rather than to another function of serine hydroxyl. First, it can be detected with a phospho-specific antibody on anillin in late cytokinesis here and previously with mass spectrometry. Second, its localization outside mitosis is unaffected by this mutation. Third, its function is partially restored with a phosphomimetic aspartic acid residue, demonstrating that negative charge at this site is important for its function, but the serine hydroxyl is dispensable. Finally, this residue is phylogenetically conserved in metazoans. Together, these data strongly support a role of phosphorylation at this site to allow anillin to function in cytokinesis.

The kinase responsible for S635 phosphorylation remains obscure. The amino acid sequence surrounding S635 does not match canonical motifs of mitotic kinases. Moreover, in preliminary experiments, we did not observe phosphorylation in the domain containing S635 by Cdk1, Plk1, or Aurora B. However, it is possible that cell contexts or modification of adjacent residues (such as Y634 phosphorylation) could alter kinase specificity. To identify the kinase and understand phospho-regulation of anillin, it will be important to consider a broad host of kinases involved in mitosis, especially those required for cytokinesis, including Rho-associated kinases.

We were unable to identify a specific mechanism by which S635 phosphorylation regulates recruitment of anillin to the equatorial membrane in anaphase. The AH-PH domain is sufficient for membrane recruitment without the region encompassing S635 [10, 16]. However, the fragment encompassing S635 could be an autoinhibitory domain, precluding AH-PH domain binding to membrane until anaphase. Phosphorylation of S635 could relieve this autoinhibitory control of AH-PH to regulate timely membrane association in anaphase.

Mechanistic and structural experiments are needed to determine if this phosphorylation controls affinity of the anillin N-terminus for efficient membrane recruitment. Moreover, it will be important to assess how phosphorylation controls membrane interaction.

One paradoxical finding is that pS635 anillin is observed primarily late in cytokinesis, yet our data suggest phosphorylation at this site is required for early anillin recruitment. One possibility is that phosphorylation of anillin occurs only late in anaphase and controls its roles in abscission [33–35]. This model is consistent with the observation of late cytokinesis failure but appears inconsistent with the impaired recruitment and the furrow instability seen with S635A. Another possible explanation for failure to detect pS635-anillin in early anaphase is that the phospho-antibody has less affinity for pS635-anillin than the total anillin antibody, so the pS635-anillin is only visualized after the ingressing furrow concentrates it in late cytokinesis. A final explanation is that phosphorylation at Y634 masks the pS635 in early cytokinesis, as we observe with phosphopeptide analysis. Thus it is possible that phosphorylation at this site occurs early in mitosis and impairs recognition until late in cytokinesis. In any case, the antibody data provide further confidence that anillin is phosphorylated at this site in human cells.

Although it may seem remarkable that many anillin phosphorylation sites appear dispensable for cytokinesis, our data do not allow for this conclusion. First, our depletion was optimized for detecting/rescuing binucleation and resulted in only ~80% knockdown of endogenous anillin; it is possible that phosphorylation on residual anillin was sufficient to retain some functions of anillin. Second, the transfection assays are heterogeneous, and it is possible that high expression in some cells masked defects of non-phosphorylatable mutants. Third, we evaluated phosphorylation sites by domain-specific mutants. Although many of the domain-specific mutants, A1-4, and A6-7, appeared to function for cytokinesis, it is possible that redundant phosphorylation sites span the boundaries we selected or, perhaps, our assay was not sensitive to subtle functions within cytokinesis. Moreover, we find that mitotic phosphorylation is decreased when Cdk1 is inactivated, possibly removing a negative regulator of anillin function in early mitosis. If so, there are additional layers of regulation as mutations of multiple putative Cdk1 phosphorylation sites did not ultimately preclude successful mitosis and cytokinesis. Additionally, phosphorylation at other sites may be important for differentially regulated functions of anillin, such as meiotic division [33].

Previously known regulatory mechanisms are insufficient to wholly explain the temporal and spatial control of anillin in cytokinesis. For example, astral microtubules [36] and Ran-GTP signals from chromatin [20], can help exclude anillin from polar membrane, although the latter appears to require close apposition of chromatin. Additionally, recruitment of anillin and RhoA to the membrane are mutually dependent [10, 18, 19], suggesting RhoA activation could be partly responsible for timing. Temporal and spatial control of the upstream kinase and anillin phosphorylation can further enhance the specificity of anillin recruitment.

In sum, using a functional screen, we identify an essential phosphorylation at S635, important to reinforce the equatorial zone of anillin. This phosphorylation site provides temporal control of its interaction with the equatorial membrane. This allows anillin to efficiently integrate Rho with its upstream regulators and downstream regulators in a timely fashion to ensure successful cytokinesis. It will be important to identify the kinase responsible for this phosphorylation and to understand how it operates with convergent mechanisms for timely and specific recruitment of anillin for cytokinesis.

## Materials and Methods

### Plasmids and Gibson Assembly

Full-length human anillin cDNA isoform 2 (accession number BC070066, Open Biosystems) was cloned into pEGFP-C1 (Clontech). This isoform was used previously to analyze anillin domain functions and encodes a protein with a 37 residue gap between the actin-binding and rho-binding domains, compared with the longest isoform. An RNAi-resistant version was made by polymerase chain reaction (PCR), by engineering of the following changes: nt 798 5’-TGCCTCTTTGAATAAA-3’ 814 to 5’-CGCAAGCTTAAACAAG-3’, creating silent mutations in the cDNA. Various derivatives were made from the RNAi-resistant template for rescue experiments. GST fusions were generated by subcloning of various anillin fragments into the pGEX-6P-1 vector (GE Healthcare).

The phosphodeficient subdomains of anillin (gBlocks: g1-g5) were generated as double-stranded, sequence-verified genomic blocks by Integrated DNA Technologies (IDT). gBlock fragments and PCR amplified anillin fragments were added to Gibson Assembly Master Mix (New England Biolabs) and incubated at 50°C for 1 h. Assembled full-length phosphodeficient mutants of anillin are then cloned into pEGFP-C1.

### Cell culture and synchronization

Cell lines were propagated at 37°C in 5% CO_2_ in media supplemented with 10% fetal bovine serum and 100 units/ml penicillin-streptomycin. The following media were used: DMEM (HeLa and ACHN), DMEM:F12 (RPE), RPMI (786-O). To enrich cells in anaphase, cells were treated with monastrol for 8 h, and fixed 60 min after release from the monastrol block.

### siRNA and DNA transfection

The following siRNA duplexes were used: control (Thermo Scientific siGENOME Non-Targeting siRNA #2 D-001206-14), anillin (40 nM; Thermo Scientific, custom order; CGAUGCCUCUUUGAAUAAA). Lipofectamine 2000 (Invitrogen) was used for siRNA/add back transfection. Cells were analyzed 48 h after transfection. For transient DNA transfection, HeLa cells were transfected using FuGENE HD (Promega) and analyzed 24 h to 48 h post transfection.

### Purification of recombinant proteins and in vitro kinase assays

GST-tagged anillin fragments were expressed in *E*. *coli* (BL21) and expression was induced by the addition of 0.1 mM IPTG at 30°C for 4–5 h. Bacteria were resuspended in PBS containing 250 mM NaCl, 10 mM EGTA, 10 mM EDTA, 0.1% Tween-20, 1 mM DTT, 1 mM phenylmethanesulfonylfluoride (PMSF) and 1 mg/ml lysozyme prior to sonication. Lysates were purified using Glutathione Sepharose 4 Fast Flow (GE Healthcare). For the in vitro kinase assay, a series of GST-tagged anillin fragments (2 μg) were incubated with each kinase in Mg^+2^-containing kinase buffer with 0.1 mM ATP and 1 μCi [γ-^32^P] ATP at 30°C for 30 min. The reaction was terminated by addition of SDS sample buffer. Samples were resolved by SDS-PAGE, visualized by Coomassie brilliant blue staining, and finally analyzed using a phosphor imager (Typhoon, GE Healthcare).

### GST pull down

HeLa cells were lysed in lysis buffer (50 mM HEPES pH 7.5, 100 mM NaCl, 0.5% NP-40, 10% glycerol, 1 mM DTT, 1 mM PMSF, protease inhibitor cocktail and phosphatase inhibitor cocktail) and incubated with purified GST-tagged anillin AH-PH at 4°C for 4 h. The beads were washed twice with lysis buffer and subjected to SDS-PAGE. Pulled down proteins were detected by immunoblotting using anti-RhoA or anti-Ect2 antibody.

### Generation of anillin antibodies

For polyclonal anillin antibodies, GST-tagged anillin amino acids 372–607 were expressed in *E*. *coli*, purified by Glutathione Sepharose 4B (GE Healthcare). Tag-cleaved proteins by Prescission protease were then used to immunize rabbits for production of antisera. Raw serum was tested for the specificity of antibodies by Western blot analysis, immunoprecipitation, and immunofluorescence staining (S5 Fig). To make phosphospecific antibodies, phosphopeptides were used to immunize rabbits. Serum was passed through a phosphopeptide affinity column. The eluted antibodies that contain a mixture of phospho- and nonphospho- antibodies were passed through a nonphosphopeptide column (Genemed Synthesis Inc.). The flow through was tested for phosphospecificity.

### Antibodies and reagents

The following primary antibodies were used: rabbit anti-anillin (1:2000; IF, 1:1000; blot), mouse monoclonal anti-GFP (1:1000; 3E6, Invitrogen), mouse monoclonal anti-cyclin B1 (1:2000; BD Biosciences), mouse monoclonal anti-β actin (1:20,000, Abcam), mouse monoclonal anti-RhoA (1:1000; 26C4, Santa Cruz Biotechnology), rabbit anti-Ect2 (1:1000, Santa Cruz Biotechnology), mouse monoclonal anti-GST (1:2000; B-14, Santa Cruz Biotechnology), mouse monoclonal anti-Flag (1:2000; M2, Sigma-Aldrich), rat monoclonal α-tubulin (1:1000; YL1/2, Millipore).

Reagents used in this study are Lambda protein phosphatase (New England Biolabs), Gibson Assembly Master Mix (New England Biolabs), nocodazole (0.2 μg/ml; EMD Biosciences), monastrol (100 μM; R&D Systems), thymidine (2.5 mM; EMD Biosciences), BI-2536 (200 nM; a gift from P. Jallepalli), ZM-447439 (4 μM; R&D Systems), RO-3306 (10 μM; R&D Systems).

### Microscopy

For immunofluorescence (IF), cells were cultured on glass coverslips in 24-well plates and fixed with 4% paraformaldehyde or ice-cold TCA for 15 min. Fixed cells were then blocked for 30 min in 3% bovine serum albumin (BSA) and 0.1% Triton X-100 in PBS (PBSTx + BSA). Primary antibodies were incubated in PBSTx + BSA for 1 h at room temperature and washed three times in PBSTx followed by secondary antibody incubation in PBSTx + BSA for 30 min at room temperature and two washes with PBSTx. Cells were counterstained with DAPI, mounted on glass slides with Prolong Gold antifade medium (Invitrogen), and allowed to cure overnight. Image acquisition was performed on a Nikon Eclipse Ti inverted microscope equipped with CoolSNAP HQ2 charge-coupled device camera (Photometrics).

To visualize GFP-tagged anillin and high resolution imaging of cytokinesis furrow formation, HeLa cells were grown in 12-well plates and transiently transfected with siRNA and anillin cDNA. 24 hr after transfection, cells were transferred to 1.5 coverslip glass-bottom, multi-well plates or 35 mm dishes (MatTek). After an additional 24 hr, cells were imaged on a Leica DMi8 inverted microscope, equipped with a 488 excitation laser, Yokogawa CSU-W1 spinning disk confocal scanner, 63x Plan Apo 1.4 NA oil-immersion objective, and controlled by Metamorph software. Environmental control was maintained using a Tokai Hit stagetop incubator. DIC and GFP images were collected every 30 seconds with a Hammatsu Orca Flash 4.0 CMOS camera. For phase contrast videomicroscopy, stable-expressing mCherry-H2B Hela were cultured and transfected as above prior. Epifluorescence and phase contrast videomicroscopy were captured on the Nikon Eclipse microscope as above with a humidified incubator to maintain cells at 37°C with 5% CO_2_. Images were captured every 5 minutes with phase contrast and mCherry epifluorescence and mitotic cells were analyzed by visualizing furrows.

Quantitative immunofluorescence was performed to evaluate GFP-Plk1 levels at equatorial membrane versus poles with regions of interest (ROIs) as shown in Figs 3C and 4H. ROIs were chosen internal to the external membrane to minimize the confounding effects of intra-versus extra-cellular levels of GFP-anillin.

### Lipid binding assay

Membrane lipid arrays (P-6002, Echelon Biosciences) were incubated with 1 μg/ml GST fusion proteins in PBS containing 0.1% Tween-20 and 3% BSA for 3 h at 25°C. After washing, proteins were detected using anti-GST antibody.

### Quantitative immunoblotting

Membranes were imaged with the Odyssey infrared imaging system (LI-COR Biosciences) and quantified using Image Studio Lite v5.2 software (LI-COR Biosciences). To quantify each band a box was drawn around the band to calculate the total pixel intensity within that box. To account for background fluorescence, a second box of equal size was drawn within the same lane and the pixel intensity of that background box was subtracted from the pixel intensity of the box containing the band of interest. Anillin intensities were normalized to β-actin for each sample, then compared to the control knockdown/addback condition to determine the percent anillin knockdown and relative total anillin expression following each transfection. The mean and standard deviation of three independent replicates are reported.

### Statistics

Replicate experiments were performed and analyzed by 2-tailed t-test with the comparisons as shown. There were no corrections for multiple t-tests. For multiple comparisons ANOVA was used as described in legends.

## Supporting Information

S1 Fig (Related to Fig 1). Cdk1 is required for anillin phosphorylation. Mitotic HeLa cells were treated with the indicated kinase inhibitors for Plk1 (BI-2536), Aurora B (ZM-447439) and Cdk1 (RO-3306) and assessed for electrophoretic shift in mitotic extracts.
(EPS)

S2 Fig (Related to Fig 4). Mutations in the A5 anillin fragment that do not alter midzone recruitment. Cells were transfected with GFP-anillin wild-type or mutants as in Fig 3 to assess ability to localize. (A) Map of the A5 fragment in anillin (left) and specific phosphorylation sites (right). (B). Transgenic GFP-tagged anillin colocalizes with total anillin. Scale bar, 10 μm.
(EPS)

S3 Fig (Related to Fig 4). S635A anillin localizes properly in interphase and early mitosis. Transiently transfected GFP-tagged wild-type (left) and S635A anillin (right) colocalize with endogenous total anillin in the nucleus (top panels) and diffusely in mitosis (bottom panels). Scale bar, 10 μm.
(EPS)

S4 Fig (Related to Fig 5). Mutation of anillin S635 causes late furrow failure. (A) To assess frequency and timing of cytokinesis failure in live cells, timelapse videomicroscopy was performed in mCherry-H2B labeled HeLa cells in which endogenous anillin was depleted and the constructs shown were transiently transfected. The merge of phase-contrast and mCherry-H2B is shown. Times are from anaphase onset and shown at upper right (minutes). Scale bar, 10 μm. (B) Graphical representation of furrow depth by time from anaphase for multiple cells for each condition.
(EPS)

S5 Fig (Related to Fig 7). Validation of phosphospecific antibody. Phospho-S635 anillin (right) is reduced upon depletion of pS635 anillin. Blot with total anillin antibody is shown at left to demonstrate depletion.
(EPS)

S6 Fig (Related to Fig 7). Distribution of phosphospecific antibody signals in immunofluorescence. Anillin and phospho-S635 anillin antibodies were used to probe cells in stages of cytokinesis with different fixation methods. pS635 stained the midbody strongly under all conditions, and was visible in mid-cytokinesis in some cells with methanol fixation. Plk1 was used as a marker to identify anaphase cells.
(EPS)

S7 Fig (Related to Fig 7). pY634 interferes with detection of pS635 by phosphospecific antibody. A dotblot was made with the specified phosphopeptides onto pre-wetted PVDF. The membrane was probed with 1:1000 pS635 antibody followed by 1:5000 anti-rabbit HRP, prior to exposure.
(EPS)

S8 Fig (Related to Figs 1–3). Validation of anillin antibody used in this study. (A) 150 kDa anillin band is reduced upon depletion. Cross-reacting band (*) is used as a loading control. (B) Antibody detects Flag-anillin after immunoprecipitation. (C) HeLa cells were transfected with full-length GFP-anillin, and then fixed and processed by immunofluorescence. In anaphase cells, the anillin band co-localizes with GFP. Two representative examples are shown. Scale bar, 10 μm.
(EPS)

S1 Video Timelapse video illustrating failure of cytokinesis with anillin knockdown and addback of GFP. Time from anaphase onset indicated in hours:min:sec.
(AVI)

S2 Video Timelapse video illustrating successful cytokinesis with anillin knockdown and addback of GFP-Anillin^WT^. Time from anaphase onset indicated in hours:min:sec.
(AVI)

S3 Video Timelapse video illustrating failure of cytokinesis with anillin knockdown and addback of GFP-Anillin^S635A^. Time from anaphase onset indicated in hours:min:sec.
(AVI)

S4 Video Timelapse video illustrating failure of cytokinesis with anillin knockdown and addback of GFP-Anillin^Y634F/S635A^. Time from anaphase onset indicated in hours:min:sec.
(AVI)

S5 Video Timelapse video illustrating failure of cytokinesis with anillin knockdown and addback of GFP-Anillin^S635D^. Time from anaphase onset indicated in hours:min:sec.
(AVI)
